# Infusion of donor feces affects the gut–brain axis in humans with metabolic syndrome

**DOI:** 10.1016/j.molmet.2020.101076

**Published:** 2020-09-08

**Authors:** Annick V. Hartstra, Valentina Schüppel, Sultan Imangaliyev, Anouk Schrantee, Andrei Prodan, Didier Collard, Evgeni Levin, Geesje Dallinga-Thie, Mariette T. Ackermans, Maaike Winkelmeijer, Stefan R. Havik, Amira Metwaly, Ilias Lagkouvardos, Anika Nier, Ina Bergheim, Mathias Heikenwalder, Andreas Dunkel, Aart J. Nederveen, Gerhard Liebisch, Giulia Mancano, Sandrine P. Claus, Alfonso Benítez-Páez, Susanne E. la Fleur, Jacques J. Bergman, Victor Gerdes, Yolanda Sanz, Jan Booij, Elles Kemper, Albert K. Groen, Mireille J. Serlie, Dirk Haller, Max Nieuwdorp

**Affiliations:** 1 Department of Internal and Vascular Medicine, Amsterdam University Medical Centers, location AMC, Amsterdam, the Netherlands; 2 Chair of Nutrition and Immunology, Technical University of Munich, Freising, Germany; 3 Department of Radiology and Nuclear Medicine, Amsterdam University Medical Centers, location AMC, Amsterdam, the Netherlands; 4 Laboratory of Endocrinology, Amsterdam University Medical Centers, location AMC, Amsterdam, the Netherlands; 5 ZIEL-Institute for Food and Health, Technical University of Munich, Freising, Germany; 6 Department of Nutritional Sciences, Molecular Nutritional Science, University of Vienna, Austria; 7 German Cancer Research Center (DKFZ), Division of Chronic Inflammation and Cancer, Heidelberg, Germany; 8 Leibniz-Institute for Food Systems Biology, Technical University of Munich, Freising, Germany; 9 Department of Laboratory Medicine, University of Regensburg, Regensburg, Germany; 10 Department of Food and Nutritional Sciences, University of Reading, Reading, United Kingdom; 11 Institute of Agrochemistry and Food Technology, Spanish National Research Council (IATA-CSIC), Valencia, Spain; 12 Department of Gastroenterology, Amsterdam University Medical Centers, location AMC, Amsterdam, the Netherlands; 13 Department of Clinical Pharmacy, Amsterdam University Medical Centers, location AMC, Amsterdam, the Netherlands; 14 Department of Endocrinology and Metabolism, Amsterdam University Medical Centers, location AMC, Amsterdam, the Netherlands

**Keywords:** Obesity, Gutmicrobiota, Gut-brain axis, Metabolites

## Abstract

**Objective:**

Increasing evidence indicates that intestinal microbiota play a role in diverse metabolic processes via intestinal butyrate production. Human bariatric surgery data suggest that the gut-brain axis is also involved in this process, but the underlying mechanisms remain unknown.

**Methods:**

We compared the effect of fecal microbiota transfer (FMT) from post-Roux-en-Y gastric bypass (RYGB) donors vs oral butyrate supplementation on (^123^I-FP-CIT-determined) brain dopamine transporter (DAT) and serotonin transporter (SERT) binding as well as stable isotope-determined insulin sensitivity at baseline and after 4 weeks in 24 male and female treatment-naïve metabolic syndrome subjects. Plasma metabolites and fecal microbiota were also determined at these time points.

**Results:**

We observed an increase in brain DAT after donor FMT compared to oral butyrate that reduced this binding. However, no effect on body weight and insulin sensitivity was demonstrated after post-RYGB donor feces transfer in humans with metabolic syndrome. Increases in fecal levels of *Bacteroides uniformis* were significantly associated with an increase in DAT, whereas increases in *Prevotella* spp. showed an inverse association. Changes in the plasma metabolites glycine, betaine, methionine, and lysine (associated with the *S*-adenosylmethionine cycle) were also associated with altered striatal DAT expression.

**Conclusions:**

Although more and larger studies are needed, our data suggest a potential gut microbiota-driven modulation of brain dopamine and serotonin transporters in human subjects with obese metabolic syndrome. These data also suggest the presence of a gut-brain axis in humans that can be modulated.

**NTR registration:**

4488.

## Highlights

**What is already known about this subject?**

• Gut microbiota are involved in human health and metabolic disease.
• Changes in gut microbiota are associated with brain disease.
• Animal studies have suggested that fecal transplantation can alter brain (dopamine and serotonin) metabolism.

**What are the new findings?**

• Donor fecal microbiota transplantation affects (^123^I-FP-CIT-determined) cerebral serotonin transporters (SERT) and dopamine transporters (DAT) in obese humans with metabolic syndrome.
• These differential changes are accompanied by alterations in plasma metabolites and fecal microbiota composition.
• New correlations between changes in microbiota strains and plasma metabolites in relation to the gut-brain axis in human obesity are observed.

**How might it impact on clinical practice in the foreseeable future?**

• This study helps to quantify the magnitude of gut microbiota-driven effects on human serotonin and dopamine in obese humans.
• This study provides a rationale for future sample size calculations for FMT trials in manipulating the gut-brain axis through gut microbiota interventions.
• This study provides a rationale for future sample size calculations for FMT trials in manipulating the gut-brain axis through gut microbiota interventions.

## 1 Introduction

The worldwide increasing prevalence of obesity and associated metabolic disorders such as type 2 diabetes mellitus (T2DM) and cardiovascular disease are of growing concern [1]. Weight loss strategies based on lifestyle interventions have little long-term success [2]. Because regulation of feeding behavior and energy metabolism is partly orchestrated in the brain, the brain's role in obesity and metabolic disturbances has been a topic of research for the past two decades. Many studies have shown a functional gut-brain axis in which gut-derived peptides, microbiota, metabolites, and neuronal feedback inform the brain about energy status [3]. These signals then elicit an appropriate feeding and metabolic response. Dopamine and serotonin are major neurotransmitters involved in these regulations. Serotonin is involved in the homeostatic regulation of body weight and food intake [4], while striatal dopamine regulates the non-homeostatic or rewarding aspects of food [5]. Recent studies linked a reduction in the cerebral serotonin transporters (SERT), dopamine transporters (DAT) and dopamine D_2/3_ receptor binding to BMI in humans [[6], [7], [8], [9], [10], [11], [12], [13], [14]]. The brain serotonergic and dopaminergic systems have also been linked to glucose regulation [15,16].

Some of these effects may be mediated by the gut-brain axis [17] driven via production of metabolites by intestinal microbiota derived from the diet [18,19]. The most compelling evidence of the role of the gut-brain axis in human metabolism comes from post-Roux-en-Y gastric bypass bariatric surgery (RYGB) studies. RYGB can alter the composition of the gut microbiota in both mice [20] and humans [[21], [22], [23]], and post-RYGB fecal microbiota transfer (FMT) in germ-free mice induced weight loss and improved glucose metabolism [20]. The difference in gut microbiota composition between obese and lean mice and humans is accompanied by variations in plasma metabolite profile [24,25], including serotonin [26,27]. Likewise, weight loss induced by RYGB significantly increased central SERT in both animals and humans [[28], [29], [30]] and striatal dopamine D_2/3_ receptor availability in humans [31]. It has recently become apparent that intestinal bacteria can regulate host serotonin metabolism [[32], [33], [34]], especially via the kynurenine pathway [[35], [36], [37]]. This pathway is also involved in the intestinal production of serotonin and dopamine and has been linked to central regulation of food intake in mice as well as intestinal passage time [38]. Another route of the gut-brain axis to modulate central control of food intake and metabolism may be via production of the short-chain fatty acid (SCFA) butyrate, which in humans is produced by intestinal bacteria from dietary fiber [39] and absorbed in the colon where it provides energy for colonic epithelial cells. It has also been shown to regulate hepatic lipogenesis and gluconeogenesis in mice on high-fat diets [40,41] and intestinal serotonin production [42,43], increasing SERT in the hypothalamus [44]. Oral butyrate supplementation affected sympathetic tone and intestinal transit times as well as physical activity and reduced liver fat in mice [45,46]. Another potential method through which the gut microbiota may influence the brain includes the bile acids. Gut microbiota synthesize secondary bile acids from primary bile acids via deconjugation and dihydroxylation, and bile acids are involved in intestinal fat absorption and regulation of glucose and energy homeostasis [47]. They function as signaling molecules through their binding potential to the nuclear receptor farnesoid X receptor (FXR) [48]. Although FXR is predominantly expressed in the liver and ileum, it can also be found in the brain, and in mice bile, acid signaling has been associated with neurological decline via upregulation of FXR [49]. Studies with FMT, a technique applied in humans for an increasing number of diseases [50], have contributed to delineate causality from association with respect to intestinal microbiota and metabolism. Animal studies showed that infusion of donor feces derived from post-RYGB mice induced weight loss and improved glucose metabolism with specific increases in bacteria involved in butyrate production [20]. Accordingly, we previously showed that infusion of feces from lean male donors in obese insulin-resistant metabolic syndrome subjects resulted in a temporary increase in insulin sensitivity and altered intestinal microbial diversity, although weight loss was not observed [51,52], whereas using feces from obese donors induced an adverse effect on recipient insulin sensitivity [53]. Regarding the former, a distinct increase in bacteria involved in butyrate production was observed, similar to results in large metagenome-wide association human studies in which a decrease in butyrate-producing bacteria was associated with T2DM [54,55]. However, we recently showed that in contrast to animal studies [56], orally administered butyrate had no effect on both insulin sensitivity and energy expenditure in human metabolic syndrome subjects [53,57]. Causality for a role of gut microbiota composition in human metabolism, probably involving the gut-brain axis, has yet to be demonstrated.

We therefore studied in a double-blind randomized controlled pilot trial whether orally administered capsules of butyrate compared to a single infusion of donor feces derived from post-RYGB patients affects brain SERT and DAT binding, whole body serotonin metabolism, and small intestinal tryptophan hydroxylase 1 (*TPH1*) gene expression as well as insulin sensitivity in subjects with metabolic syndrome. As secondary endpoints, we correlated these outcomes with changes in gut microbiota composition and plasma metabolites. We also studied changes in MRI intrahepatic triglycerides (IHTG), sympathetic activity, and intestinal transit time in these subjects.

## 2 Methods

### 2.1 Human experiment

#### 2.1.1 Study subjects

Treatment-naïve omnivorous Caucasian male or post-menopausal female subjects (n = 24) aged 50–70 years with metabolic syndrome were recruited via local advertisements. Subjects were included if they fulfilled the National Cholesterol Education Program (NCEP) criteria for metabolic syndrome (≥3/5: fasting plasma glucose (FPG) ≥ 5.6 mmol/l and/or homeostatic model assessment of insulin resistance (HOMA-IR) ≥ 2.5, triglycerides ≥ 1.7 mmol/l, waist circumference > 102 cm (males)/> 88 cm (females), HDL-cholesterol ≤ 1.04 mmol/l (m)/≤ 1.30 mmol/l (f) and blood pressure ≥ 130/85 mmHg). HOMA-IR was calculated from fasting plasma insulin (FPI) and glucose: FPG ∗ (FPI ∗ 6.945)/22.5. Subjects were excluded if they had used any medication in the prior 3 months. Other exclusion criteria were pre- or probiotic supplementation, substance abuse (nicotine, drugs, or alcohol > 2 units/day), eGFR < 60 ml/min, contraindication for MRI, unstable weight, or history of a cardiovascular event or psychiatric disorder. As fecal donors, 6 otherwise healthy Caucasian males and post-menopausal females aged 50–70 years who lost >30% of their body weight 1 year after RYGB and did not use any medication (barring vitamins) were selected and recruited by their treating physician at the Bariatric Surgery Clinic at the former Slotervaart Hospital in Amsterdam. They completed questionnaires regarding their dietary and bowel habits, travel history, comorbidity including family history of diabetes mellitus, and medication use. They were screened for the presence of infectious diseases as previously published [58]. Male donors donated to males and female donors to females, and donors could donate to multiple recipients. This study was conducted at the Amsterdam University Medical Centers (UMC), Academic Medical Center, in accordance with the Declaration of Helsinki (updated version 2013) and CONSORT guidelines. All of the participants provided written informed consent and all of the study procedures were approved by the institutional review board (IRB) (ethics committee) of the Amsterdam UMC (Academic Medical Center). This study was prospectively registered at the Dutch Trial registry (https://www.trialregister.nl/trial/4488). Patients were not invited to comment on the study design and were not consulted to develop relevant patient outcomes or interpret the results. Patients were not invited to contribute to the writing or editing of this document for readability or accuracy. Data quality and patient safety were monitored by the Clinical Research Unit staff at the AMC.

#### 2.1.2 Study design

In this double-blind randomized controlled intervention trial, metabolic syndrome subjects were randomized (using computerized randomization) to receive either a single autologous fecal transplantation, serving as placebo, followed by 4 g of oral sodium butyrate tablets (Sensilab, Poland) once daily for 4 weeks, which was the maximum daily dose allowed by the IRB based on a previous human intervention study [59] (butyrate group, n = 12) or a single post-RYGB donor fecal transplantation followed by similar daily amounts of placebo tablets (similar tablet composition except for butyrate content produced by Sensilab, Poland) for 4 weeks (post-RYGB FMT group, n = 12) (Supplementary Figure 1). Compliance was evaluated by counting the number of capsules returned after 4 weeks of treatment. At baseline and after 4 weeks, all of the measurements were conducted, including a hyperinsulinemic euglycemic clamp (HIEC), brain magnetic resonance imaging (MRI), single-photon emission computed tomography (SPECT) imaging, ^1^H-liver magnetic resonance spectroscopy (MRS), sympathetic activity using a plethysmography-based blood pressure measurement device and small intestinal biopsies (for *TPH1* expression). Fasting plasma, 24 h urine (for 5-hydroxyindoleacetic acid [5-HIAA] levels), and morning feces were also collected at these time points. All the participants completed an online nutritional diary (https://mijn.voedingscentrum.nl/nl/eetmeter) to monitor their caloric intake of carbohydrates, fat, protein, and fibers. Physical activity energy expenditure (PAEE) was measured via an accelerometer (ActiHeart; CamNTech Ltd., Cambridge, UK), and intestinal transit time was assessed using Sitzmark capsules as previously described [53]. For a detailed description of the study design, see the online supplementary methods.

### 2.2 Measurements

#### 2.2.1 Fecal transplant procedure

FMT was performed as previously described [53]. Each subject received a single FMT at baseline, either autologous or allogenic as determined via a double-blinded randomization procedure. On the treatment day, the donor delivered fresh morning stools to the hospital. Each study subject received a duodenal tube via gastroscopy and underwent colon lavage with 3–4 l of Klean-Prep (macrogol) by a duodenal tube. Donor feces were diluted in 500 mL of 0.9% saline solution and filtered through cotton gauze. This produced a 500 ml filtrate used for the FMT, which each subject received 2 h after bowel lavage through the duodenal tube via a 50 cc syringe.

#### 2.2.2 Two-step HIEC and resting energy expenditure (REE)

REE was measured in all of the subjects during the final 20 min in both the basal state and HIEC by indirect calorimetry. Oxygen consumption and carbon dioxide production were continuously measured for 20 min using a ventilated hood system (Vmax Encore 29; SensorMedics, Anaheim, CA, USA). REE was then calculated from each subject's oxygen consumption and carbon dioxide production [60]. A 2-step HIEC clamp was used to measure insulin sensitivity [61]. After an overnight fast, the subjects visited the clinical trial unit, where they received 2 catheters in the peripheral veins of both arms. One catheter was used to infuse [6,6-^2^H_2_]glucose and [1,1,2,3,3-^2^H_5_]glycerol tracers (99% enriched; Cambridge Isotopes, Andover, MA, USA), glucose 20% enriched with [6,6-^2^H_2_]glucose to approximate plasma enrichment, and insulin (Actrapid; Novo Nordisk Farma, Alphen aan de Rijn, the Netherlands). The other catheter was used to sample each subject's blood, which was arterialized by heating the arm with a heated hand box at 57 °C. Two hours before starting the clamp (t = −2 h), a primed continuous infusion of both [6,6-^2^H_2_]glucose and [1,1,2,3,3-^2^H_5_]glycerol was started and continued until the end of the experiment. After 2 h (t = 0), 3 samples for glucose, glucose and glycerol enrichment, free fatty acids (FFAs), and glucoregulatory hormones were sampled, and the first clamp step was started by infusing insulin at a rate of 20 mU·m^−2^ (body surface area)·min^−1^. Plasma glucose was measured every 10 min using a glucose analyzer (YSI 2300 Stat Plus Glucose Lactate Analyzer, YSI Life Sciences, Yellow Springs, Ohio, USA). To maintain plasma glucose at 5 mmol l^−1^, 20% glucose enriched with [6,6-^2^H_2_]glucose was infused at a variable rate. Insulin infusion was increased after 2 h of insulin infusion (t = 2 h) to 60 mU·m^−2^·min^−1^ for the second clamp step. At t = 2 and 4 h, 5 blood samples were obtained to assess glucose and glycerol enrichment, free fatty acids (FFAs), and glucoregulatory hormones. [6,6-^2^H_2_]glucose and [1,1,2,3,3-^2^H_5_]glycerol enrichment was measured as previously described [62,63]. The rates of appearance (Ra) of glucose and glycerol and rates of disposal (Rd) of glucose were subsequently calculated using the modified forms of the Steele equations for (non-)steady state measurements as previously described [53,61,64]. Hepatic insulin sensitivity was calculated as the percentage suppression of basal endogenous glucose production (EGP) by insulin during the first step of the clamp.

#### 2.2.3 Systemic hemodynamics

Blood pressure and central hemodynamics were assessed via a Nexfin device (Edwards Lifesciences, Irvine, CA, USA) that utilizes the volume-clamp method to measure blood pressure and heart rate, while cardiac output was estimated using the CO-trek algorithm [65]. Heart rate variability, a marker of sympathovagal balance, was determined by calculating the standard deviation of the normal-to-normal intervals (SDNN, inter-beat intervals derived from continuous blood pressure recordings after filtering) [66]. Non-invasive continuous finger arterial blood recordings were obtained in the supine position for 10 min after 10 min of rest. All the analyses were conducted in a blinded fashion.

#### 2.2.4 MRI for anatomical mapping of the brain and ^1^H MRS for determining IHTG content

A T1-weighted MRI scan of the brain was performed on each individual (for anatomical reference to determine diencephalic SERT and striatal DAT) on a 3.0 T Philips Ingenia scanner (Philips Healthcare, Best, the Netherlands) with a 16-channel head coil.

IHTG was measured via ^1^H-MRS conducted on the same scanner using a 26-channel torso coil. First, T1-weighted coronal and axial localizer images of the abdomen were obtained that were then used to position a 20 × 20 × 20 mm voxel. Because the diaphragm, edges of the liver, or other vascular and biliary structures must be avoided, the voxel was placed in the right hepatic lobe. The acquisition time and voxel size were standardized for all the subjects. Spectra were obtained using first-order iterative shimming, a point-resolved spectroscopy (PRESS) sequence with repetition time/echo time (TR/TE) = 2000/35 ms, and 64 signal averages during free breathing. The liver ^1^H-MR spectra were evaluated using jMRUI software. Water non-suppressed spectra were used to quantify the lipid signal resonances. The relative fat content was expressed as a ratio of the fat peak area over the cumulative water and fat peak areas (1.3 ppm/([1.3 ppm +4.65 ppm]). Calculated peak areas of water and fat were corrected for T2 relaxation. The IHTG percentage was determined as previously described [67]. All of the analyses were conducted in a blinded fashion.

#### 2.2.5 Brain SPECT imaging

**Figure 1:**
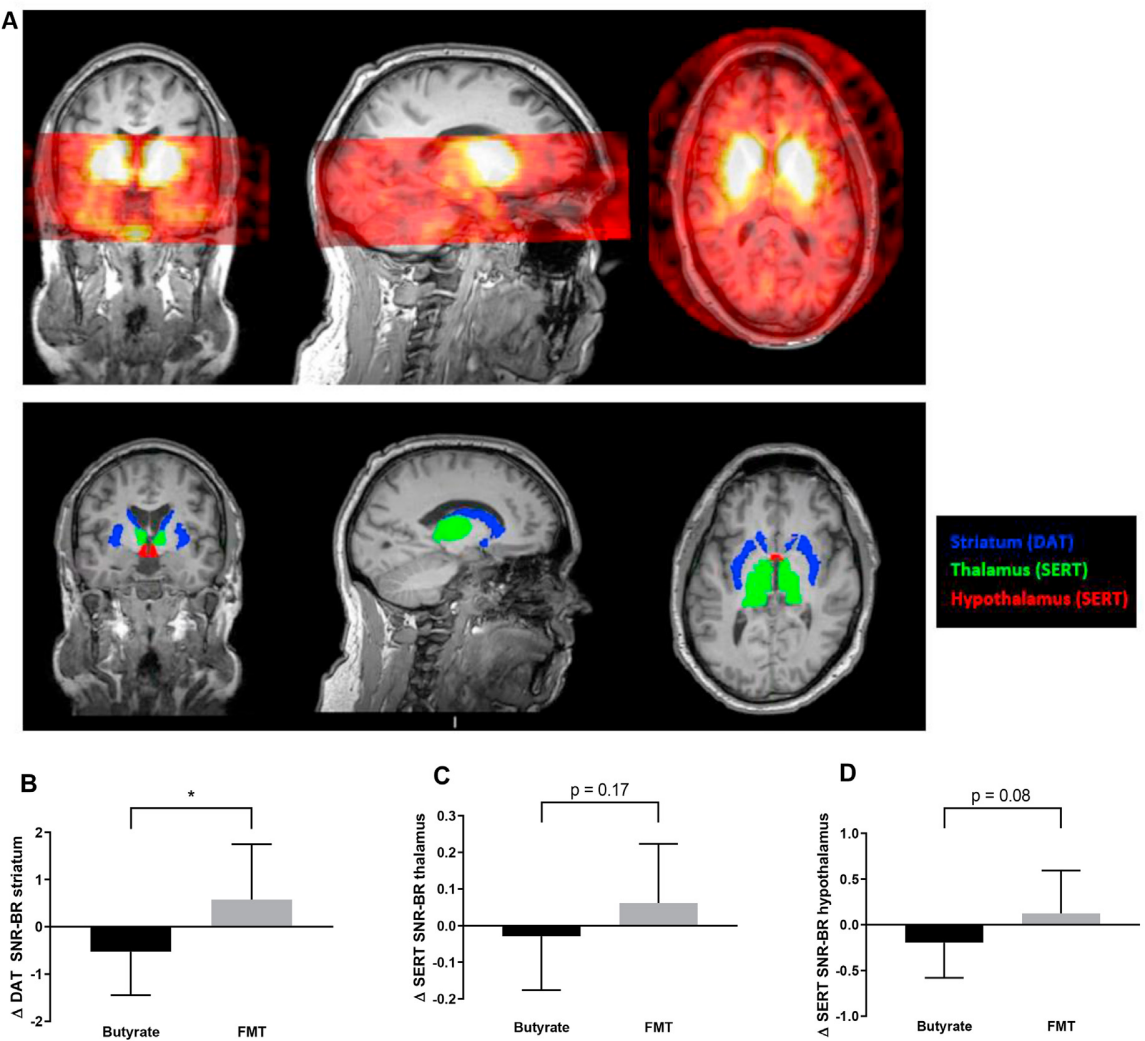
**A. Brain SPECT image and ROIs.** The top panel shows a representative example of a brain MRI overlaid with a brain SPECT image obtained 2 h after the intravenous administration of the radioligand ^123^I-FP-CIT. In the bottom panel, the regions of interest (ROIs) for specific parts of the human brain are shown, which were subsequently used to determine SERT binding in the thalamus and hypothalamus and DAT binding in the striatum. B, C, and D. Changes in striatal DAT as well as SERT binding in thalamus and hypothalamus. Changes in DAT and SERT binding between group comparisons showed that striatal DAT-binding ratios decreased in the butyrate group and increased in the FMT group, resulting in a significant change over time between both groups, while for SERT binding, a trend was observed. ∗p < 0.05.

Each subject underwent SPECT imaging of the brain 2 and 3 h after intravenous administration of well-validated radioligand ^123^I-FP-CIT at a total dose of 115 MBq (range 110–120 MBq; specific activity > 750 MBq/nmol; radiochemical purity > 98%, produced according to the GMP criteria at GE Healthcare, Eindhoven, the Netherlands) [68]. This tracer bound to DAT in the striatum and extra-striatal SERT, which was optimally visualized and quantified in the SERT-rich (hypo)thalamic region of the brain 2 h after injection and in the DAT-rich striatum 3 h after injection [69,70]. Figure 1A shows an example. The subjects were scanned after an overnight fast and pretreated with potassium iodide for thyroid blockade of free radioactive iodide. For scanning, the Inspira HD system was used, a brain-dedicated tomographic SPECT scanner (Neurologica, Boston, MA, USA), and an acquisition protocol as previously described (slice thickness 4 mm and acquisition time 180 s/slice) [71]. All the scans were reconstructed in 3D mode and corrected for attenuation. One SPECT scan could not be performed in 1 subject in the butyrate group after treatment due to technical problems with the scanner. On the SPECT scan day, the subjects scored their hunger and appetite on a visual analog scale (VAS) as previously described [72].

#### 2.2.6 ROI analysis

To determine SERT binding in the thalamus and hypothalamus and DAT binding in the striatum, a region of interest (ROI) analysis was conducted as previously described [71] (Figure 1A). Striatal and thalamic masks were obtained from individual T1-weighted MRI scans using FreeSurfer (version 5.3.0.) [73, 74]. Hypothalamic masks were then manually drawn on the MRI scans, a reliable method [75], via ITK-SNAP (version 3.4.0, PICSL, University of Pennsylvania, PA, USA) using anatomical landmarks as previously described [76]. Activity in the cerebellum was assumed to represent non-displaceable binding (non-specific binding and free radioactivity). Individual cerebellar masks were obtained in the T1-weighted space by warping the cerebellum (without vermis) as described in the Harvard–Oxford subcortical atlas (http://fsl.fmrib.ox.ac.uk/fsl/fslwiki/Atlases) to the individual T1w MRI using FSL (FMRIB Software Library, version 5.0.6, Oxford, UK). All the analyses were conducted in a blinded manner. All the ROIs were then registered and resliced to the SPECT scan using statistical parametric mapping. A specific to non-specific binding ratio (SNS-BR) was calculated as (mean ROI-binding - mean non-specific cerebellar binding)/mean non-specific cerebellar binding), which was used as the outcome measure (binding potential, BP_ND_).

#### 2.2.7 Laboratory analysis

Plasma biochemistry measurements were performed as described earlier [57]. Plasma bile acid concentrations were determined using an LC-MS/MS system as previously described [77]. Fecal short-chain fatty acids (SCFAs) (butyrate, propionate, and acetate) were determined in fresh morning fecal samples by HPLC as previously described [78]. Twenty-four hours urine was collected at baseline and after 4 weeks to assess the concentration of the main metabolite of serotonin 5-HIAA as a measurement of the whole body serotonin levels as previously reported [79].

#### 2.2.8 Fecal intestinal microbiota analysis

The subjects' DNA was extracted at CISC using a QIAamp Fast DNA Stool Mini Kit (Qiagen, Hilden, Germany) according to the manufacturer's instructions with a prior bead beating step in 2 mL micro centrifuge tubes containing 0.1 mm diameter glass beads, ∼200 mg faces, and 1 mL InhibitEX buffer. Bead beating was carried out in a Mini-Bead Beater apparatus (BioSpec Products, Bartlesville, OK, USA) with two cycles of shaking for 1 min and incubation on ice between cycles. The fecal DNA was measured via UV methods (NanoDrop, Thermo Fisher Scientific, Wilmington, DE, USA), and an aliquot of every sample was prepared at 20 ng/μl with nuclease-free water for polymerase chain reactions (PCRs). The V3–V4 hypervariable regions of the 16S ribosomal ribonucleic acid (rRNA) gene were amplified using 20 ng of DNA (1 uL diluted aliquot) and 25 PCR cycles consisting of the following steps: 95 °C for 20 s, 55 °C for 20 s, and 72 °C for 20 s. Phusion High-Fidelity Taq Polymerase (Thermo Fisher Scientific, Wilmington, DE, USA) and S-D-Bact-0341-b-S-17 (CCTACGGGNGGCWGCAG) and S-D-Bact-0785-a-A-21 (GACTACHVGGGTATCTAATCC) primers that target a wide range of bacterial 16S rRNA genes (Klindworth et al., 2013) were used during PCR. The primers were 6-mer barcoded. Dual barcoded PCR products consisting of ∼480bp were purified from triplicate reactions with an Illustra GFX PCR DNA and Gel Band Purification Kit (GE Healthcare, Little Chalfont, UK) and quantified through Qubit 3.0 and a Qubit dsDNA HS Assay Kit (Thermo Fisher Scientific, Waltham, MA, USA). The samples were multiplexed by combining equimolar quantities of amplicon DNA (100 ng per sample) and sequenced in an Illumina MiSeq platform with a 2 × 300 PE configuration (Centro Nacional de Análisis Genómico, CNAG, Barcelona, Spain). Sample de-multiplexing was carried out using sequence information from the respective DNA barcodes using the Je software suite (v.2.0) allowing no errors in the indexes. Primers were removed from the sequences using Cutadapt (v.2.8). The resulting reads were then processed using USEARCH (v.11.0.667) [80]. Forward and reverse reads were merged allowing for a maximum of 30 mismatches in the overlapping region. Merged reads were discarded if they were shorter than 350 bp or longer than 500bp. Remaining contigs were filtered using an expected error-based quality filter as described by Edgar et al. [81]. Filtered contigs were dereplicated and unique sequences were denoised using the UNOISE3 algorithm to infer Amplicon sequence variants (ASVs). All the merged reads were subsequently mapped against the resulting ASVs to produce a count table. Taxonomy was assigned to the ASVs using the USEARCH sintax algorithm [82] using the SILVA (v.123) database. The sample counts were rarefied to 17,391 counts per sample. For downstream machine-learning models, ASVs were further filtered by abundance, keeping only ASVs that had on average at least 3 counts per sample (372 ASVs). The raw amplicon sequencing microbiome data from this study will be deposited in the European Nucleotide Archive repository.

#### 2.2.9 Plasma metabolomics

Plasma samples (350 μL) were mixed with D2O at a 1:1 volume ratio. All the homogenized samples were centrifuged (10 min, 4 °C, and 13,000 rpm) and transferred to 5 mm NMR tubes for analysis by NMR spectroscopy. The NMR experiments were conducted at the Chemical Analysis Facility (CAF, University of Reading, Reading, UK) using a Bruker AV700 NMR instrument working at 700.19 MHz equipped with a 5 mm inverse TCI CryoProbe for increased sensitivity (Bruker BioSpin, Rheinstetten, Germany). A standard 1-dimensional NOESY-PR-1D experiment was conducted on all the samples followed by a Carr-Purcell-Meiboom-Gill (CPMG) experiment. Both methods applied a sequence to pre-saturate the water peak. The CPMG experiment was used to reduce the signal contribution from albumin and lipoproteins present in the plasma and improve the detection of signals from smaller molecules. All the samples were analyzed at 300 K. A 65k data point spectrum (spectral width 14705 Hz) was obtained by recording 128 scans following 8 dummy scans. The spectral phase and baseline corrections were assessed using MestreNova software (version 10.0 Mestrelab Research, Santiago, Spain). The NMR spectra were referenced to glucose at 5.23 ppm.

#### 2.2.10 Duodenal biopsy analysis

Duodenal biopsies were obtained at baseline and after 4 weeks for gut microbiota analyses as well as *TPH1* expression [83]. Low bacterial load present in the duodenal biopsies complicated the retrieval of enough bacterial DNA for standardized PCR procedures and 16S amplicon sequencing. Therefore, the duodenal microbiota could not be analyzed. mRNA was isolated using an RNA isolation protocol optimized for (very small) biopsies. In short, biopsies were mixed with 300 μl TriPure (Roche, Basel, Switzerland) and homogenized on ice using a sterile RNAse-free pestle. After brief centrifugation, 60 μl of chloroform was added. The samples were then added to a Heavy Phase Lock gel tube (Quanta Bio, Beverly, MA, USA) and centrifuged (15 min, 12.000×*g*, and 4 °C). The aqueous phase was transferred and mixed with 1 volume of 70% ethanol. The mixture was added to an RNeasy MinElute spin column (QIAgen, Tegelen, the Netherlands). RNA was washed according to the manufacturer's protocol and eluted in 14 μl RNAse-free water. The RNA concentration was measured using NanoDrop 1000 (Thermo Fisher Scientific, Landsmeer, the Netherlands). From the RNA, cDNA was produced according to the manufacturer's protocol using a SensiFAST cDNA Synthesis Kit (Bioline, London, UK). The expression of h*TPH1* and h*36B4* genes was measured using qPCR (CFX 384, BioRad, Hercules, CA, USA). Per reaction, 1 μl of 10 μM Primer Mix (h*TPH1* forward: 5′-CCCGCTTTTGGCTGAACCTA-3′ and h*TPH1* reverse: 5′-AGTAGCACGTTGCCAGTTTTT-3′ or h36B4 forward: 5′-ACGGGTACAAACGAGTCCTG-3′, and h36B4 reverse: 5′-GCCTTGACCTTTTCAGCAAG-3′), 1 μl RNAse-free water, 5 μl of SensiFAST SYBR No-ROX (Bioline, London, UK), and 3 μl cDNA (5 ng/μl) was used. The h*TPH1* gene expression was normalized using *36B4* as a housekeeping gene. For histology, enterochromaffin cells were stained for serotonin. In short, 10x images were taken with a Leica DM microscope. An ROI was set in the images by a trained pathologist. ImageJ software was used to quantify the amount of positive serotonin cells per 100 μM2 as previously described [84].

#### 2.2.11 Power calculation and statistical analysis

The primary endpoints were changes in hypothalamic SERT and striatal DAT binding, small intestinal *TPH1* expression, serotonin staining, and urinary 5-HIAA levels in relation to peripheral and hepatic insulin sensitivity, VAS appetite, and hunger scores upon treatment. Secondary endpoints were changes in fecal short-chain fatty acids and plasma bile acids, changes in dietary intake, REE, and physical activity energy expenditure in relation to changes in gut microbiota composition and plasma metabolites. Changes in the intestinal passage time (Sitzmark capsules), sympathetic tone, and IHTG were determined before and after the intervention. The sample size was based on previous studies in which we found a 30% change in the binding ratio of ^123^I-FP-CIT to striatal DAT and SERT in the diencephalon upon intervention (0.65–0.46 with SD 0.15) [85]. Based on this finding, a sample size calculation was performed with a two-sided significance level of 0.05 and power of 80% showing that 10 subjects per arm were needed. Taking into account a 10–20% dropout rate, we included 12 subjects in each arm for a total of 24 subjects.

Depending on the distribution, the data are presented as mean ± standard deviation or median (interquartile range) and tested when paired (within group change) with either the paired Student's t test or Wilcoxon's signed-rank test and when unpaired (between 2 groups) with the unpaired Student's t test or Mann–Whitney U test. A p value < 0.05 was considered statistically significant. Spearman's rank correlation coefficient was used for correlation analysis.

#### 2.2.12 Machine-learning model

To identify which intestinal bacterial species were the most discriminative between both groups, we applied the extreme gradient boosting classification algorithm combined with stability selection [86]. We applied the extreme gradient boosting (XGBoost) machine-learning classification algorithm [87] combined with stability selection to identify which variables had the best discriminative power in predicting the treatment groups. This technique was used on the microbial composition data (ASVs) and plasma metabolite levels (NMR peak values). To predict the treatment groups, we used the relative change (delta) of each variable between baseline and end time point in separate models for the metabolomics and microbiota dataset. Each analysis produced a ranked list of the most important variables. Variable importance was calculated using the permutation feature importance of the model. The permutation feature importance is defined as the decrease in a model score when a single feature value is randomly shuffled [88]. This procedure severs the relationship between the feature and target, so the decline in the model score is indicative of how much the model depends on the feature. While the variables identified by the classification model frequently lead to statistically significant results, they can also be unstable. In our approach, we addressed this problem via the stability selection procedure coupled with the model selection [86]. Variable stability was reflected in the frequency that a particular variable was identified in multiple simulations in a re-randomized dataset. Stability selection was performed by randomly subsampling 80% and the model was then tested on the remaining 20% of the data not used in the training. This was repeated 100 times. Receiver operating characteristics area under the curve (ROC AUC) scores were computed each time and averaged for the final test ROC AUC. A permutation (randomization test) was used to evaluate statistical validity of the results. In the permutation test, the outcome variable was randomly reshuffled 1,000 times while the corresponding omics profiles were maintained intact [89]. We used Python v3.7 (www.python.org) with Numpy, Scipy, and Scikit-learn [90] packages to implement the machine-learning model and 10-fold cross-validation to estimate optimal hyperparameters.

## 3 Results

### 3.1 Participants’ baseline characteristics

Between 2015 and 2017, we included 24 treatment-naïve subjects with metabolic syndrome aged between 50 and 70 years who were randomized to receive either an autologous (placebo) FMT and daily oral ingestion of butyrate for 4 weeks (butyrate group, n = 12) or allogenic (post-RYGB donor) FMT and placebo capsules (FMT group, n = 12). In this regard, FMT donors 2, 4, and 5 were used twice, donors 3 and 6 were used once, and donor 1 was used three times. The baseline patient characteristics are presented in Table 1 and Supplementary Table 1. All of the subjects were insulin resistant (homeostasis model assessment for insulin resistance; HOMA-IR ≥ 2.5). Six post-RYGB surgery donors were included, 3 males and 3 females, who were matched by sex to the subjects in the allogenic FMT group (Supplementary Table 1). Both treatments, daily oral ingestion of butyrate and a single FMT, were well tolerated and no side effects or serious adverse events occurred (Supplementary Table 2). Compliance was verified in all of the subjects and no differences were found between the 2 groups. There were no significant changes in body weight, most hemodynamic measurements, and dietary intake (Table 1, Table 6). However, HbA1c, total cholesterol, and triglycerides significantly decreased after butyrate treatment, whereas HbA1c was significantly decreased upon allogenic post-RYGB FMT (Table 1). No effect on body weight or glucose metabolism was observed after donor FMT. Finally and in line with our previous studies [53,57], fecal SCFAs and bile acids after either oral butyrate or post-RYGB FMT did not significantly change (Table 5).

**Table 1:** Basic and metabolic parameters.

	Butyrate (n = 12)			Donor FMT (n = 12)			
	Before	After	P	Before	After	P	∗P
BMI, *kg/m*^*2*^	34.3 ± 3.4	34.2 ± 3.7	0.76	33.0 ± 3.5	33.1 ± 3.4	0.35	0.36
Fasting glucose, *mmol/l*	6.1 [5.7–6.3]	5.7 [5.4–6.5]	0.23	5.6 [5.2–5.8]	5.5 [5.0–5.9]	0.281	0.54
Fasting insulin, *pmol/l*	70 [59–114]	61 [48–96]	0.25	77 [53–105]	66 [62–83]	0.169	0.81
HbA1c, *mmol/l*	40 [35–45]	37 [34–44]	**0.04**	37 [34–39]	35 [33–38]	**0.04**	0.41
Cholesterol							
- Total, *mmol/l*	5.5 ± 1	5.1 ± 0.8	**0.04**	5.9 ± 1.2	5.6 ± 1	0.09	0.85
- LDL, *mmol/*	3.5 [2.6–4.3]	3.2 [2.8–4]	0.14	3.9 [3.1–4.5]	3.7 [2.8–4.2]	0.27	0.72
- HDL, *mmol/l*	1.4 [1.2–1.6]	1.4 [1.2–1.5]	0.55	1.6 [1.3–1.8]	1.4 [1.2–1.6]	0.05	0.26
- Triglycerides, *mmol/l*	1.4 [1.1–1.7]	1.2 [0.9–1.4]	**0.03**	1.2 [0.8–1.5]	1.3 [0.9–1.4]	0.84	0.11
Glucose Rd, *umol/kg.min*	38.5 [30.8–47.4]	38.1 [27.5–42.9]	0.21	38.1 [33.4–53.2]	39.9 [27.9–45.9]	0.21	0.32
EGP suppression, *%*	74.8 [64.8–82.4]	79 [70.2–86.7]	0.87	82.7 [75.7–97.3]	91.2 [73.8–99.7]	0.34	0.81
REE, *kcal/day*	1761 ± 259	1738 ± 210	0.56	1694 ± 220	1673 ± 205	0.63	0.91
Glycerol Ra suppression, *%*	66.6 [63.4–69.6]	68.6 [47.8–74.4]	0.58	64.7 [56.8–67.6]	53.5 [44.8–67]	0.31	0.60
FFA suppression, *%*	89.6 ± 4.9	86.9 ± 5.9	0.09	89.3 ± 7.7	90.1 ± 5.2	0.68	0.15
IHTG, *%*	4.7 [2.8–11.1]	4.3 [3.5–12.0]	0.91	5.8 [3.9–12.9]	6.1 [3.3–11.8]	0.59	0.77
CRP, *mg/ml*	2.5 [2.9–5.0]	1.7 [0.9–4.6]	0.38	2.1 [0.9–3.1]	3.2 [1.0–5.2]	0.13	0.10
Creatinine, *μmol/l*	76 ± 12	76 ± 11	0.82	75 ± 14	74 ± 13	0.67	0.66
SBP, *mmHg*	134 [100–141]	128 [124–141]	0.35	126 [115–140]	119 [111–138]	0.69	0.45
DBP, *mmHg*	72 [60–75]	70 [65–77]	0.43	73 [66–75]	68 [63–78]	0.209	0.25
MAP, *mmHg*	95 [76–98]	91 [87–102]	0.34	91 [85–103]	85 [82–102]	0.34	0.22
HR, *beats/min*	61 [56–67]	58 [55–63]	0.12	62 [60–70]	63 [59–66]	0.93	0.16
CO, *l/min*	5.5 [5.2–6.1]	6 [4.9–6.3]	0.53	5.5 [5.1–6.1]	5.5 [5.4–6]	0.21	0.48
SDNN, *millisecond*	44 [34–55]	64 [50–73]	**0.01**	49 [41–57]	54 [40–70]	0.53	0.13
Compliance, *number of capsules not taken*	0	0		0	0		

Data are expressed as mean ± SD or median [IQR]. ∗p = change over time between groups. A p value < 0.05 was considered statistically significant. BMI = body mass index, HbA1c = glycated hemoglobin, HDL = high-density lipoprotein, HOMA-IR = homeostatic model assessment of insulin resistance, LDL = low-density lipoprotein, CRP = c-reactive protein, SBP = systolic blood pressure, DBP = diastolic blood pressure, EGP = endogenous glucose production, Rd = rate of disappearance (insulin sensitivity), Ra = rate of appearance, FFA = free fatty acid, REE = resting energy expenditure, IHTG = intrahepatic triglyceride, MAP = mean arterial pressure, HR = heart rate, CO = cardiac output, SDNN = standard deviation of the normal-to-normal intervals (inter-beat intervals derived from continuous blood pressure recordings after filtering).

**Table 2:** DAT and SERT binding.

	Butyrate			FMT			
	Before	After	P	Before	After	P	∗P
Striatal DAT binding (SNR-BR)	5.21 ± 1.26	4.71 ± 0.87	0.08	4.60 ± 1.22	5.18 ± 1.27	0.11	**0.02**
Thalamic SERT binding (SNR-BR)	0.46 ± 0.15	0.42 ± 0.15	0.54	0.45 ± 0.18	0.51 ± 0.16	0.21	0.17
Hypothalamic SERT binding (SNR-BR)	0.51 ± 0.26	0.32 ± 0.23	0.12	0.41 ± 0.31	0.53 ± 0.5	0.37	0.08

Data are expressed as mean ± SD. ∗p = change over time between groups. A p value < 0.05 was considered statistically significant. DAT = dopamine transporter, SERT = serotonin transporter, SNR-BR = specific to non-specific binding ratio.

**Table 3:** Systemic and duodenal serotonin metabolism.

24 h urine	Butyrate, n = 12			FMT, n = 12			∗P
	Before	After	P	Before	After	P	
5-HIAA per 24h, *μmol/l*	30 [18–42]	29 [17–34]	0.27	42 [31–74]	33 [25–46]	0.35	0.93
duodenal EC cells, *cells/100 uM*^*2*^	1.2 [1.0–1.7]	1.1 [1.0–1.6]	1.00	1.1 [0.9–1.6]	1.2 [0.9–1.4]	1.00	0.90
Small intestinal biopsy	n = 7			n = 7			
*TPH1* expression, *relative to 36B4*	0.0027 [0.0013–0.0035]	0.0037 [0.0013–0.0135]	0.49	0.0044 [0.0017–0.0272]	0.0016 [0.0010–0.0053]	0.17	0.22

Data are expressed as median [range]. ∗p = change over time between groups. 5-HIAA = 5-hydroxyindoleacetic acid (24 h urine), EC = enterochromaffin, TPH1 = tryptophan hydroxylase 1 (qPCR duodenal cells).

**Table 4:** VAS appetite and hunger scales.

	Butyrate			FMT			∗P
	Before	After	P	Before	After	P	
VAS appetite, *cm*	33 [6–60]	28 [10–49]	0.62	43 [14–68]	49 [24–78]	0.21	0.22
VAS hunger, *cm*	33 ± 24	27 ± 21	0.42	26 ± 27	31 ± 27	0.44	0.25

Data are expressed as mean ± SD and median [range]. ∗p = change over time between groups. VAS = visual analogue score.

**Table 5:** SCFA and BA metabolism.

	Butyrate			FMT			∗P
	Before	After	P	Before	After	P	
Fecal SCFAs							
- Acetic acid, *umol/g*	448 [298–648]	454 [172–755]	0.72	320 [163–575]	320 [163–576]	0.06	0.16
- Butyric acid, *umol/g*	94 [59–129]	92 [41–145]	0.86	96 [55–133]	66 [35–96]	0.07	0.55
- Propionic acid, *umol/g*	191 [124–281]	196 (104–283]	0.53	167 [97–225]	132 [96–167]	0.53	0.53
Plasma BAs							
- Total, *μM*	1.03 [0.77–1.85]	1.04 [0.72–1.44]	0.24	1.83 [0.92–2.46]	1.53 [1.07–3.53]	0.69	0.60
- Primary, *μM*	0.515 [0.463–0.841]	0.53 [0.312–0.655]	0.18	0.847 [0.371–1.511]	0.85 [0.447–2.317]	0.53	0.16
- Secondary, *μM*	0.504 [0.383–0.799]	0.488 [0.38 7–0.708]	0.27	0.629 [0.459–1.522]	0.622 [0.365–1.015]	0.18	0.81
- Conjugated, *μM*	0.645 [0.459–0.957]	0.647 [0.400–0.817]	0.31	1.032 [0.462–1.746]	0.768 [0.625–0.817]	0.53	1.00
- Unconjugated, *μM*	0.411 [0.249–0.766]	0.359 [0.284–0.568]	0.24	0.524 [0.356–0.88]	0.476 [0.26–0.689]	0.530	0.73

Data expressed as median [range]. ∗p = change over time between groups. SCFA = short-chain fatty acid, BA = bile acid.

**Table 6:** Energy expenditure.

	Butyrate			FMT			∗P
	Before	After	P	Before	After	P	
Excreted Sitzmarks, *number*	35 [21–45]	44 [36–53]	0.18	40 [30–52]	41 [23–54]	0.53	0.12
AEE, *kcal/day*	908 [540–1281]	818 [588–1016]	0.32	925 [433–1301]	750 [371–1187]	0.15	0.95
Caloric intake, *kCal/day*	1682 [1493–2015]	1664 [1492–1803]	0.1	1761 [1381–2034]	1777 [1372–2194]	0.24	0.05
Fat intake, *g*	64 [60–83]	62 [54–65]	0.26	68 [54–82]	68 [53–98]	0.19	0.10
Protein intake, *g*	85 [75–95]	79 [64–96]	0.19	93 [74–104]	92 [83–100]	0.58	0.64
Fiber intake, *g*	17 [14–22]	14 [12–19]	0.38	16 [13–19]	17 [14–19]	0.34	0.19
Carbohydrate intake, *g*	165 [143–209]	155 [137–210]	0.51	160 [128–198]	188 [137–206]	0.11	0.17

Data are expressed as median [range]. ∗p = change over time between groups. AEE = active energy expenditure.

### 3.2 Effect of donor FMT vs butyrate on brain SERT and DAT

After 4 weeks of treatment, a decrease after butyrate treatment and an increase after post-RYGB FMT in striatal DAT binding was observed (p = 0.02) (Table 2 and Figure 1A). Heart rate variability (expressed as SDNN) significantly increased 4 weeks after oral butyrate but not after post-RYGB donor FMT treatment (Table 1). VAS appetite and hunger scales did not change before and after 4 weeks of treatment (Table 4). A positive trend was observed in brain (hypo)thalamic SERT binding ratios in the FMT group (Table 2 and Figure 1B,C). We found no changes in 5-HIAA or duodenal *TPH1* mRNA expression and enterochromaffin cell serotonin staining (Table 3).

### 3.3 Effect of donor FMT vs butyrate on insulin sensitivity

In line with our previous studies [53,57], no effect of either post-RYGB donor FMT or oral butyrate on hepatic insulin sensitivity (EGP suppression) and peripheral insulin sensitivity (Rd) was observed (Table 1 and Supplementary Figure 2). No significant effect was observed on glucoregulatory hormones (Supplementary Table 3) or insulin action in adipose tissue, determined as the suppression of the glycerol rate of appearance and IHTG content (Table 1). In line with our previously published results, no significant differences after either intervention in active energy expenditure as measured via an accelerometer and intestinal transit time were observed (Table 6).

### 3.4 Fecal microbiota and plasma metabolites after post-RYGB donor FMT compared to butyrate correlated with DAT

**Figure 2:**
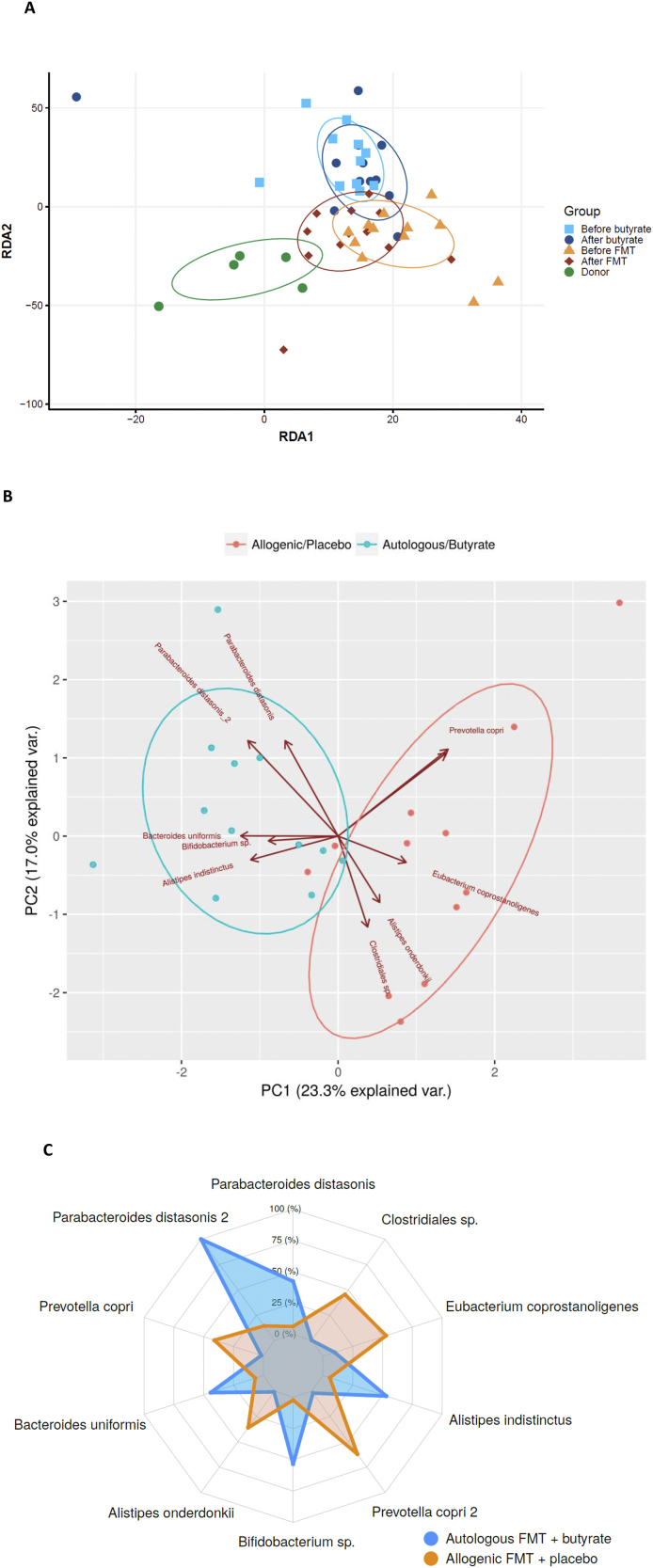
**Changes in fecal microbiota composition after butyrate supplementation or allogenic FMT.** (A) Biplot of redundancy analysis (RDA axis 1 vs axis 2) of fecal microbiota data constrained by butyrate vs allogenic FMT treatment variables (before, baseline, and 4 weeks after treatment). Baseline fecal microbiota composition in the post-RYBG feces donors is also shown. (B) Biplot of deltas from the top 10 amplicon sequence variant (ASV) markers of fecal microbiota after allogenic FMT and butyrate treatment. AUC = 0.83 ± 0.29. (C) Spider plot depicting a panel of bacterial species that significantly differentiated between the 2 different treatment groups based on changes in the fecal microbiota composition. The axis reflects the amount of change in % of the bacterial species after either treatment.

**Figure 3:**
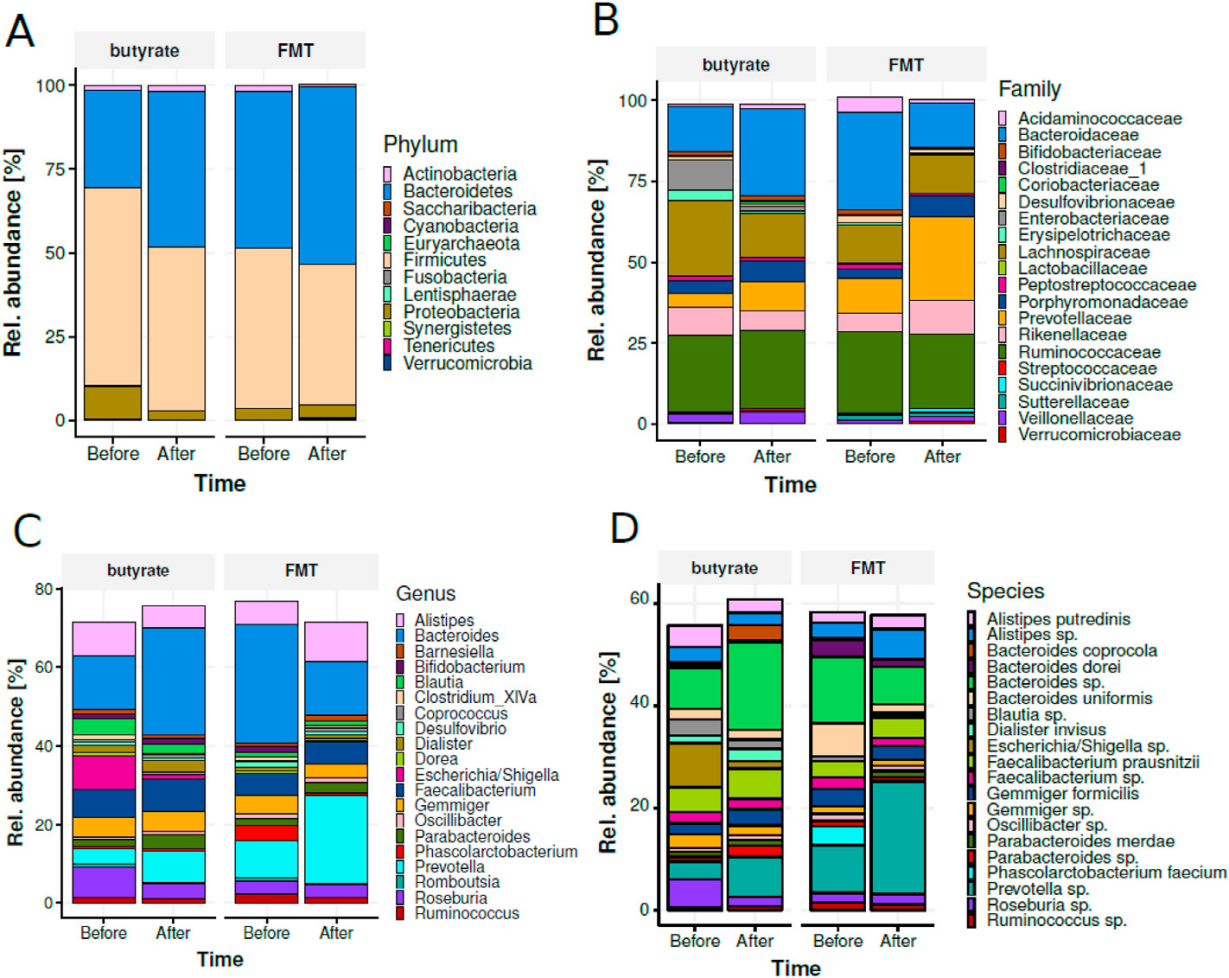
**Gut microbiota composition.** Gut microbiota composition at phylum (A), family (B), genus (C), and species (D) levels stratified by time and group of subjects.

**Figure 4:**
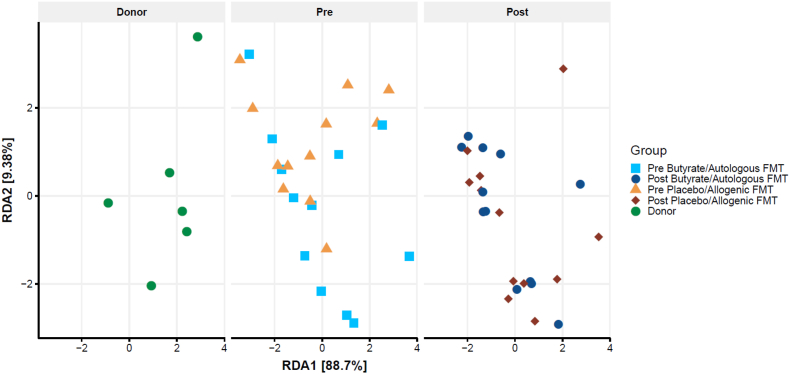
**Changes in plasma metabolites.** Biplot of redundancy analysis (RDA axis 1 vs axis 2) of the plasma metabolite data constrained by butyrate and allogenic FMT treatment variables (before, baseline, and 4 weeks after treatment). Baseline plasma metabolites in the post-RYBG feces donors is also shown. The brackets on the axis labels refer to the proportion of the constrained variance explained by the respective axis/RDAs. In this case, the constrained variance was 7.8% of the total variance.

**Figure 5:**
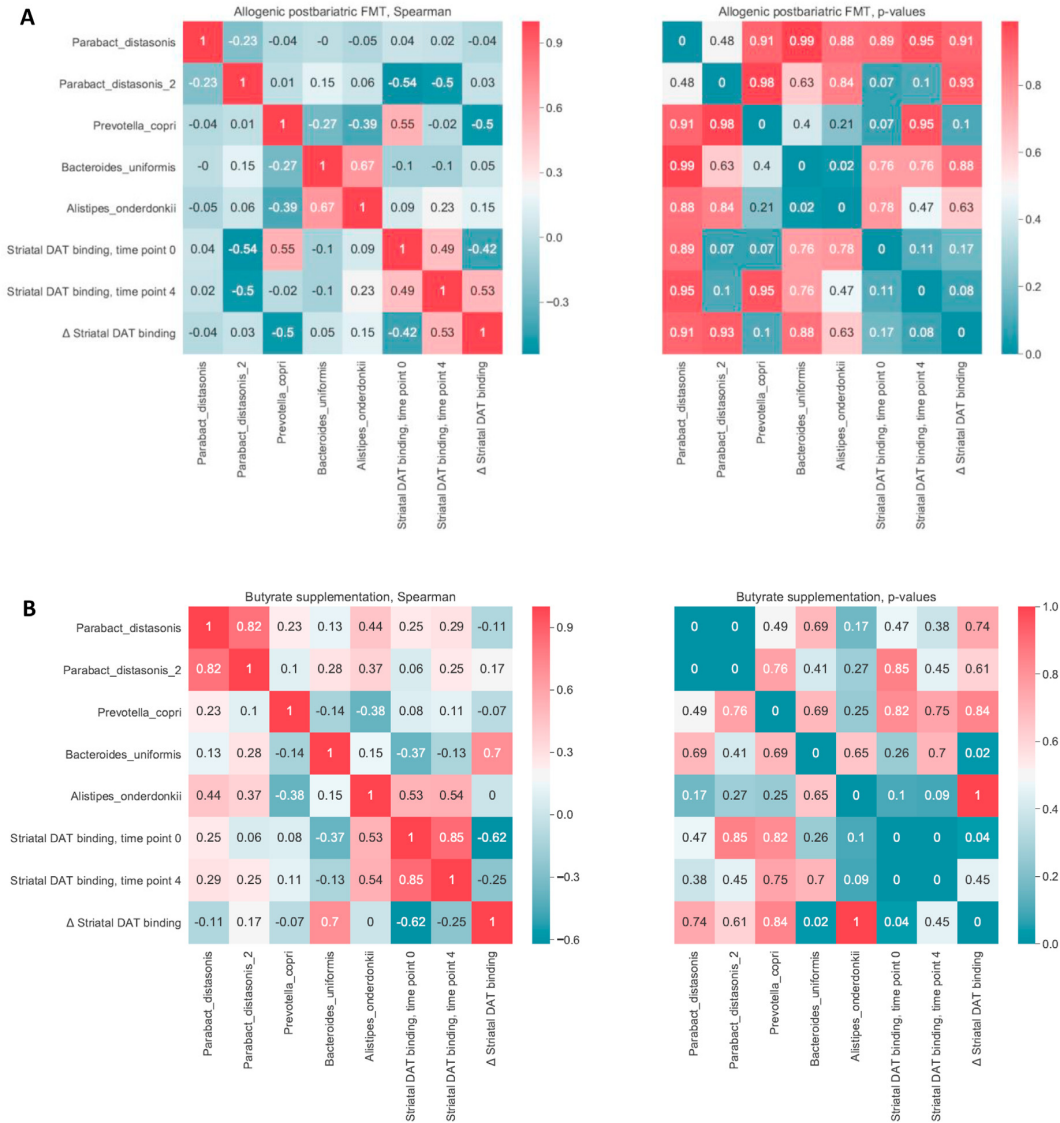
**Correlations between changes in gut microbiota and striatal DAT binding.** (A) Allogenic post-RYGB FMT donor group (left panel: Spearman's correlation values, right panel: p values). Changes in *Prevotella copri* correlated inversely with changes in DAT binding (rho = −0.50, p value = 0.1). (B) Butyrate intervention group (left panel: Spearman's correlation values; right panel: p values). Changes in *Bacteroides uniformis* positively correlated with changes in DAT binding (rho = 0.70, p value = 0.02).

**Figure 6:**
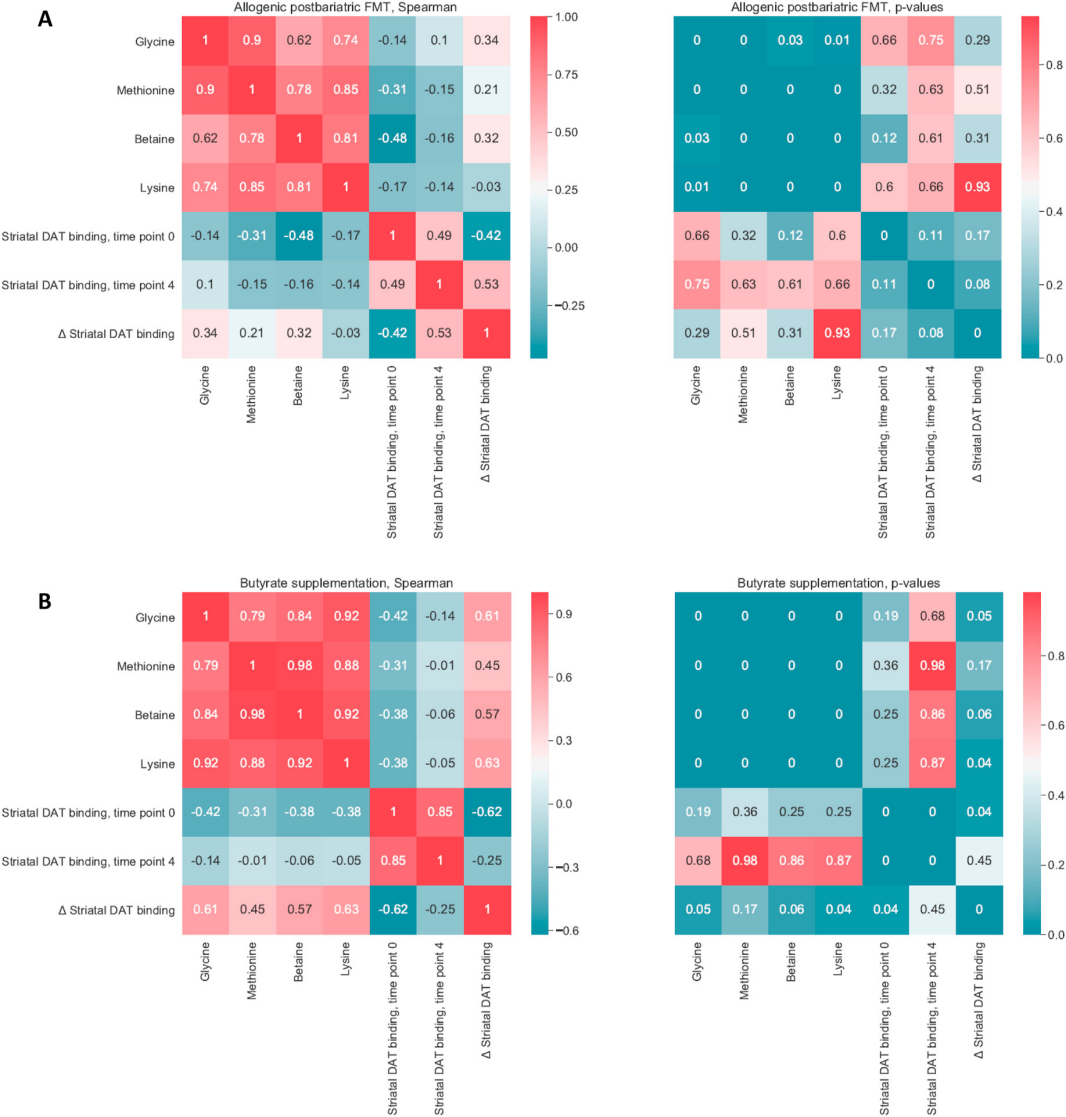
**Correlations between changes in plasma metabolites and change in striatal DAT binding.** (A) Allogenic post-RYGB FMT donor group (left panel: Spearman's correlation values, right panel: p values). Changes in betaine and glycine correlated positively with change in DAT without reaching significance. (B) Butyrate intervention group (left panel: Spearman's correlation values, right panel: p values). All 4 metabolites correlated positively with changes in DAT with glycine (rho = 0.61, p value = 0.05) and lysine (rho = 0.63, p value = 0.04).

**Figure 7:**
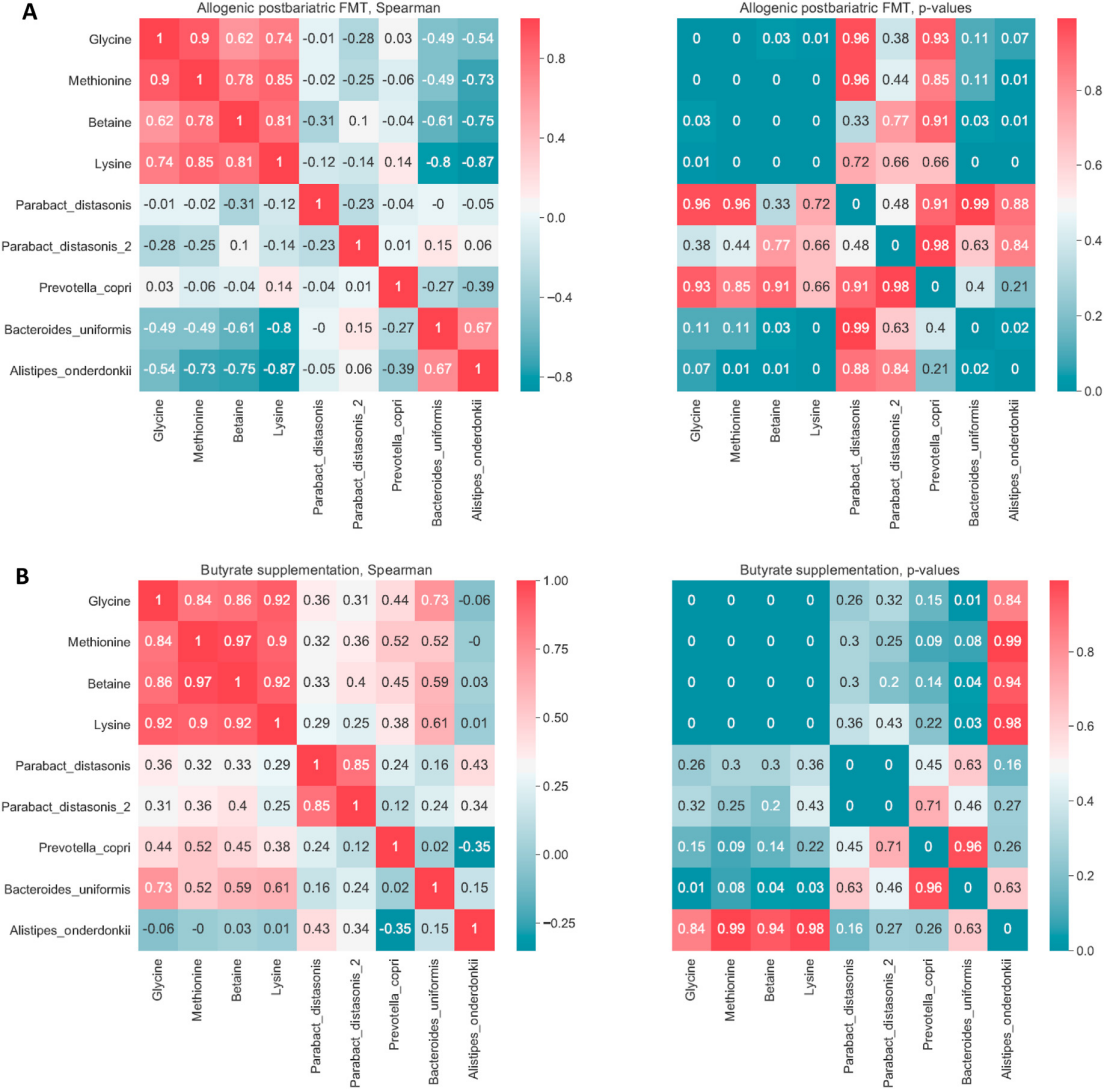
**Correlations between changes in plasma metabolites and gut microbiota.** (A) Allogenic post-RYGB FMT donor group (left panel: Spearman's correlation values, right panel: p values). *Bacteroides uniformis* correlated negatively with all 4 metabolites with betaine (rho = −0.61, p value = 0.03) and lysine (rho = 0.8, p value = 0.001). (B) Butyrate intervention group (left panel: Spearman's correlation values, right panel: p values). *Bacteroides uniformis* correlated significantly with plasma glycine, betaine, and lysine (p = 0.01, p = 0.04, and p = 0.03) and a positive trend was observed with *Prevotella copri*.

After post-RYGB donor FMT, we observed a shift in fecal microbiota composition toward the donors’ composition (Figure 2A). The gut microbiota composition of the donors is shown in Supplementary Figure 3. We found that fecal microbiota including *Parabacteroides distasonis*, *Clostridiales* sp., *Eubacterium coprostanoligenes*, *Alistipes*, *Prevotella copri*, *Bifidobacterium* sp., and *Bacteroides uniformis* were altered after post-RYGB donor FMT, as shown in a PCA plot as a qualitative visualization (Figure 2B), while treatment with oral butyrate resulted in a change in, among others, *Bacteroides uniformis* (Figures 2C and 3). Regarding changes in fasting plasma metabolites, after allogenic FMT, a shift was observed toward the FMT donor plasma metabolite profile (Figure 4), altering plasma lysine, glycine, methionine, and betaine (Supplementary Figure 4). Increases in fecal levels of *Bacteroides uniformis* were significantly associated with an increase in DAT (r = 0.7, p < 0.05), whereas increases in *P. copri* showed an inverse association (r = −0.5, p = 0.1) (Figure 5). Moreover, with regard to correlations between changes in plasma metabolites and striatal DAT binding ratios, in the butyrate treatment group, changes in plasma glycine and lysine levels significantly correlated with DAT (Figure 6B), whereas plasma betaine and glycine correlated with DAT after FMT (Figure 6A). Changes in *Bacteroides uniformis* were significantly inversely correlated with changes in plasma betaine and lysine after FMT (Figure 7A), whereas upon oral butyrate, changes in *Bacteroides uniformis* showed a significant linear correlation with changes in plasma glycine, betaine, and lysine (Figure 7B).

## 4 Discussion

In line with our previous studies [53,57], in the current randomized controlled trial, no significant effect of either daily oral butyrate or donor feces derived from post-RYGB patients was observed in the treatment-naïve human subjects with metabolic syndrome on either body weight or insulin sensitivity as measured by the gold-standard HIEC. However, the (albeit modest) significant change in human brain striatal DAT between both groups after 4 weeks was associated with alterations in both gut microbiota composition and plasma metabolites involved in the methionine/*S*-adenosylmethionine (SAMe) cycle. Interestingly, this pathway is known to be essential for neurotransmitter synthesis such as dopamine and serotonin. Collectively, these data suggest that gut microbiota play a role in the human brain dopaminergic system and therefore might affect food intake. Whether this is mediated via vagus nerve signaling [91] and plasma metabolites involved in the SAMe pathway remains to be determined. Thus, although the interventions did not result in clinically significant changes, they are hypothesis generating. DAT is a presynaptic membrane protein expressed in dopaminergic terminals that regulates synaptic and extracellular dopamine by facilitating its re-uptake into presynaptic terminals. This enables the important process of fine-tuning dopamine signaling, which is essential in reward processing and behavioral learning [92]. Changes in DAT expression are associated with neurological and psychiatric disorders such as ADHD, autism, and Parkinson's disease (PD) [93]. In line with our findings, previous studies in PD patients (who were characterized by an altered striatal DAT expression due to nigrostriatal dopaminergic degeneration) have shown altered fecal *P. copri* levels compared to controls [94], underscoring the role of the gut-brain axis and more specific the enteric nervous system in human disease [95,96]. In this respect, our observation of an altered heart rate variability after treatment with butyrate also suggests the involvement of vagal nerve signaling in the modulation of the gut-brain axis via changes in microbiota. This is in line with accumulating data in animals pointing toward the influence of the gut microbiota on motivational behavior through the vagal nerve [91,97]. Vagal afferents can be activated by gut endocrine cell secretion of serotonin gut peptides and also directly by gut microbiota-derived metabolites such as SCFA butyrate [98]. The vagus nerve projects to the nucleus solitarius of the brainstem. This nucleus then projects to many brain regions, including the paraventricular nucleus (PVN) of the hypothalamus and the ventral tegmental area in the midbrain [99]. These projections could play a role in the observed changes in striatal DAT and might be important pathways in the microbiota gut-brain food intake as well as glucose metabolism axis, but more detailed studies are necessary to explore these pathways in humans.

Although we observed a positive trend in (hypo)thalamic SERT binding in the FMT group, we found no significant changes in 24 h 5-HIAA secretion, indicative of whole body serotonin concentrations. Serotonin staining in EC cells and the *TPH1* mRNA expression in the duodenal tissue biopsies before and after the intervention were unchanged in both groups. However, other plasma metabolites including lysine, glycine, methionine, and betaine significantly changed after treatment, all correlating with changes in DAT binding, more markedly upon treatment with butyrate. In this regard, Colosimo et al. [100] recently showed that microbiota-derived amine-based neurotransmitter receptor agonists including lysine affect histamine receptor H4 (HRH4) signaling in the brain. Although lysine, an essential amino acid, has previously been associated with reduced anxiety and stress response in both animals and humans [101] mediated through changes in serotonin in the central amygdala [102] and gut [103] in rats, to the best of our knowledge, our study is the first to report a link with dopamine via DAT. Methionine, betaine, also known as trimethylglycine, and glycine are all involved in one-carbon (C1) metabolism and the SAMe cycle [104], which are interrelated with dopamine and serotonin metabolism. In humans, betaine and methionine are dietary methyl donors from which SAMe can be synthesized, which is the body's main methyl donor, found in all tissues but particularly liver cells and essential to numerous cellular methyl transfer reactions such as DNA methylation (crucial in epigenetics) and the formation of neurotransmitters, including serotonin and dopamine [105]. Unsurprisingly, low SAMe has been linked to mood disorders characterized by dopamine and serotonin depletion such as major depression [106,107]. Indeed, depressed rats had lower SAMe compared to controls, which increased after treatment with probiotics (lactic acid bacteria *Lactobacillus helveticus* R0052 and *Bifidobacterium longum* R0175), while elevated plasma dopamine in the depressed rats was lowered [108].

Our randomized controlled trial also has certain limitations, including the small sample size study executed in Caucasian subjects only and thus needs confirmation in a larger RCT including subjects with different ethnicities [109]. As dietary intake was stable, the observed effects on the brain were most likely driven by altered intestinal microbiota. However, using a single donor FMT might not be enough to induce a durable effect, although in one of our previous studies, multiple FMTs did not have any significant metabolic effects in metabolic syndrome subjects [52]. Second, due to ethical (radiation exposure) constraints, we were only able to determine SERT and DAT on 2 occasions, whereas a 12-week time point would have been a valuable addition to study the long-term effects. We did not assess the effect of our interventions on food reward-related outcomes such as food behavior-related questionnaires or MRI imaging with computer calculations. Nevertheless, our intervention study provides further evidence for the existence of a microbiota gut-brain axis in humans possibly involving the striatal dopaminergic system.

## 5 Conclusion

This study demonstrated that modulating gut microbiota composition or increasing one of its major metabolites intraluminally affects striatal DAT binding in humans. These changes were associated with alterations in *Bacteroides uniformis* and *Prevotella* spp. abundance as well as with metabolites involved in the methionine/*S*-adenosylmethionine (SAMe) cycle, an important pathway in neurotransmitter synthesis. Although our results are still mainly hypothesis generating due to the small sample size, we speculate that the gut milieu affects vagal afferents projecting to the brain stem, resulting in modulation of neuronal dopaminergic circuits involved in hedonic regulation of feeding behavior.

## Author contributions

A.V.H., A.K.G., D.H., and M.N. designed the study. A.V.H., V.S., A.S., D.C., G.M.D., M.T.A., S.R.H., M.W., A.M., I.L., A.N., I.B., M.H, A.D., A.J.N., G.L., G.M., S.P.C., A.B–P., J.J.B., V.G., Y.S., J.B., E.K., and M.J.S. conducted the research. A.V.H., S.I., A.P., and E.L. performed the statistical analysis. A.V.H., A.K.G., D.H., and M.N. drafted the paper. All of the authors critically reviewed the manuscript.

## Funding

This study was funded by FP7-EU consortium MyNewGut grant agreement no. 613979 (to which A.V.H. was appointed). M. Nieuwdorp is supported by a ZONMW-VIDI grant 2013 (016.146.327).
