# Supplementary material for: Infusion of donor feces affects the gut–brain axis in humans with metabolic syndrome
Source: Mol Metab. 2020 Sep 8;42:101076. doi: 10.1016/j.molmet.2020.101076 (PMC7536740; doi:10.1016/j.molmet.2020.101076)
Supplement: Multimedia component 1 [file mmc1.docx]

**Supplementary tables and figures Hartstra et al.**

**Supplementary Figure 1. Study design**


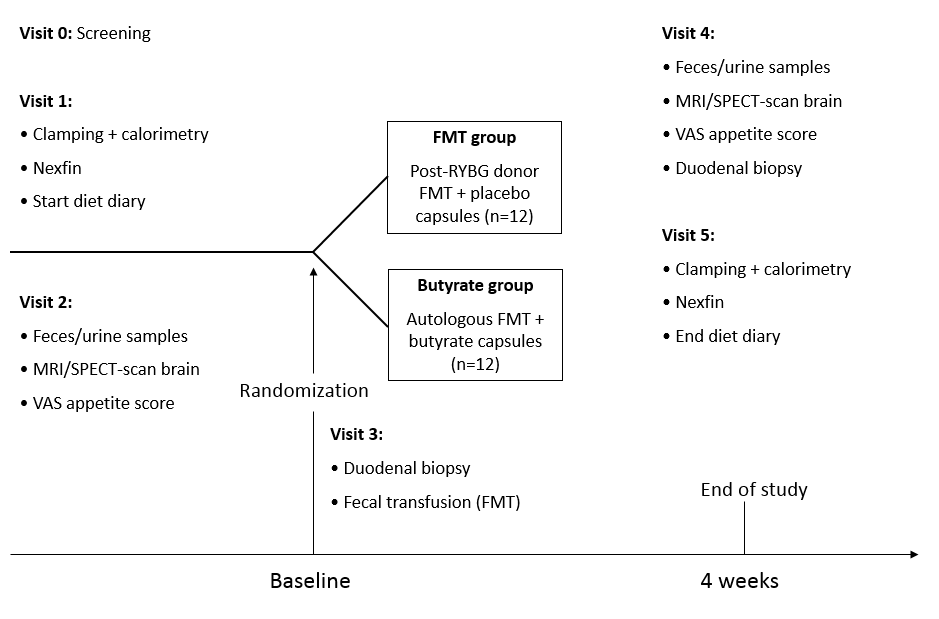


**Supplementary Table 1. Baseline characteristics of the participants**

|  | **Butyrate** | **FMT** | **FMT donors** |
| --- | --- | --- | --- |
| N | 12 | 12 | 6 |
| Sex (M/F) | 7/5 | 6/6 | 3/3 |
| Age (y) | 60.6 ± 7.8 | 60.2 ± 6.4 | 53.5 [52.8-57.8] |
| Weight (kg) | 102.6 ± 7.9 | 98.0 ± 10.7 | 99.5 [62.5-110.2] |
| BMI (kg/m^2^) | 34.3 ± 3.4 | 33.0 ± 3.5 | 28.2 [22.9-35.5] |
| Fasting glucose (mmol/l) | 6.1 [5.7-6.3] | 5.6 [5.2-5.8] | 5.1 [4.9-5.1] |
| HOMA-IR | 3.6 ± 1.4 | 3.0 ± 0.9 | 1.0 [0.8-1.4] |
| HDL (mmol/l) | 1.4 [1.2-1.6] | 1.6 [1.3-1.8] | 1.8 [1.5-2.5] |
| SBP (mmHg) | 134 [100-141] | 126 [115-140] | 134 [118-152] |
| DBP (mmHg) | 72 [60-75] | 73 [66-75] | 76 [69-86] |

**Supplementary Table 2. Side effects and bowel habits**

|  | **Butyrate** | | **FMT** | |
| --- | --- | --- | --- | --- |
|  | Week 0 | Week 4 | Week 0 | Week 4 |
| IBS ROME III criteria | 0 | 0 | 0 | 0 |
| IBS-QOL | 34 | 34 | 34 | 34 |
| Side effects |  |  |  |  |
| - Nausea | 0 | 0 | 0 | 0 |
| - Flatulence | 0 | 0 | 0 | 0 |
| - Cramps | 0 | 0 | 0 | 0 |
| - Borborygmi | 0 | 0 | 0 | 0 |
| - Gastric reflux | 0 | 0 | 0 | 0 |

**Supplementary Figures 2A, B, C, and D. Hepatic (EGP suppression) and peripheral (Rd) insulin sensitivity**

| **Supplementary Table 3. Circulating insulin levels during clamp tests at weeks 0 and 4** | | | | | | |
| --- | --- | --- | --- | --- | --- | --- |
|  | **Basal state** | | **Step 1** | | **Step 2** | |
|  |  |  |  |  |  |  |
|  | Butyrate | FMT | Butyrate | FMT | Butyrate | FMT |
| **Clamp week 0** |  |  |  |  |  |  |
| Insulin, *pmol/l* | 70 [59-114] | 77 [53-105] | 272 [212-323] | 322 [256-354] | 737 [614-814] | 772 [740-868] |
| Glucagon, *ng/l* | 87 [80-105] | 72 [65-100] | 70 [65-79] | 68 [55-81] | 67 [59-77] | 54 [48-74] |
| Cortisol, *ng/l* | 180 [164-234] | 200 [150-279] | 212 [192-248] | 244 [178-313] | 203 [168-292]ᵇ | 176 [155-203] |
| **Clamp week 4** |  |  |  |  |  |  |
| Insulin, *pmol/l* | 61 [48-96] | 66 [62-83] | 249 [204-314] | 323 [272-346] | 663 [589-866] | 794 [714-888] |
| Glucagon, *ng/l* | 74 [66-111] | 81 [59-93] | 68 [62-98] | 61 [53-80] | 60 [52-86] | 51 [47-70] |
| Cortisol, *ng/l* | 192 [155-213]ᵃ | 255 [180-289]ᵃ | 244 [185-293] | 248 [218-339] | 183 [140-253]ᵇ | 182 [121-256] |

**Supplementary Figures 3A, B, C, and D. Gut microbiota composition of post-RYBG donors**


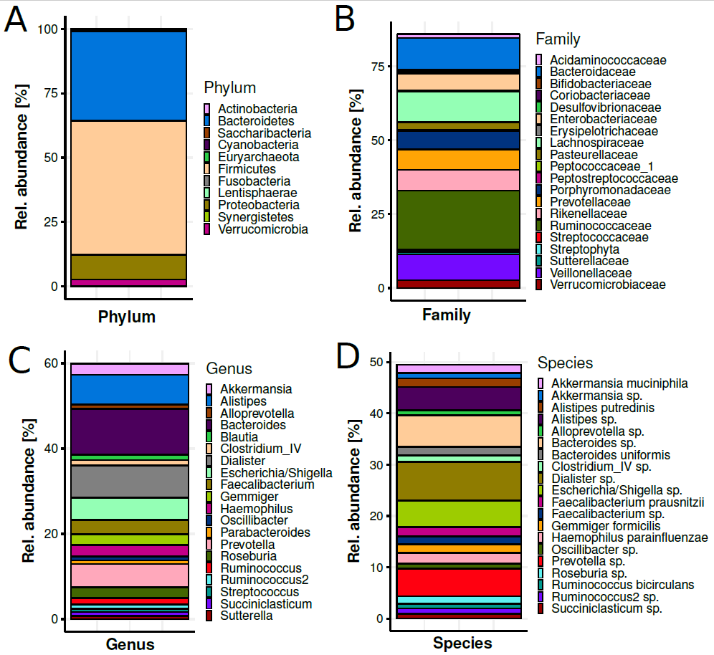


**Supplementary Figures 4A and B. Heat maps depicting significant associations between plasma and 24 h urine metabolites per treatment group**

1. **Changes in plasma metabolites B. Changes in urine metabolites**


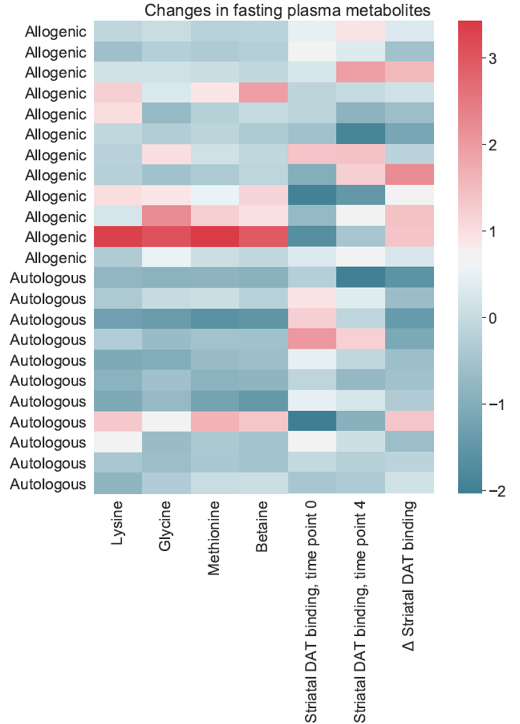

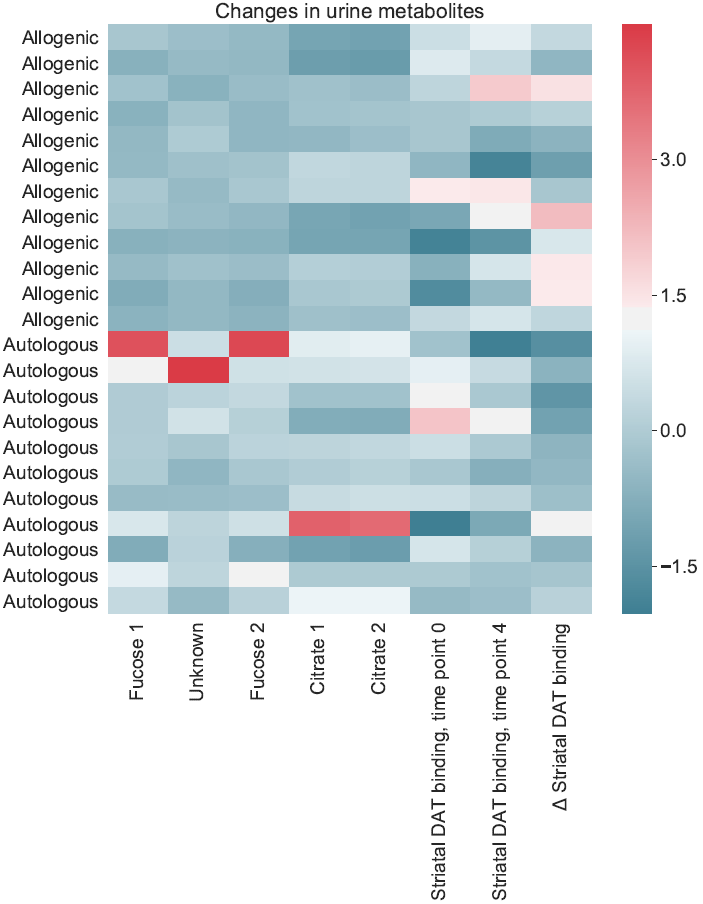


**Supplementary methods (Hartstra et al.)**

**Supplementary methods**

*Study design*

After inclusion, the subjects came to our clinical research facility at the Amsterdam UMC, location AMC for 3 baseline visits executed within one week, all after an overnight fast (Supplementary Figure 1). On the first study day, the subjects underwent a 2-step HIEC with stable isotopes ^2^H_2_-glucose and [1,1,2,3,3-^2^H_5_]glycerol to measure their endogenous glucose production (EGP), hepatic insulin sensitivity (percentage suppression of EGP under hyperinsulinemic conditions), peripheral (rate of disposal, Rd) insulin sensitivity, suppression of glycerol rate of appearance (Ra, a measure of adipose tissue insulin sensitivity), and FFA suppression (suppression of circulating plasma FFA relative to basal state). During the clamp, the resting energy expenditure (REE) was assessed using indirect calorimetry and sympathetic activity via Nexfin.

On study day 2, after oral pretreatment with potassium iodide tablets, the subjects received radioligand [^123^I]FP-CIT as an intravenous bolus. After 2 and 3 h, a SPECT scan of the brain was performed to assess binding to SERT and DAT in the diencephalon and striatum, respectively. Individual SPECT images were combined with individual brain MRIs registration and delineation of ROIs. During this MRI session, the liver was also scanned to detect any changes in liver fat. To assess satiety, hunger and appetite were scored on a visual analog scale (VAS).

On study day 3, a gastroduodenoscopy was performed for duodenal biopsies that were immediately collected in sterile tubes, snap-frozen in liquid nitrogen, and processed as previously described^1^. This was directly followed by duodenal tube placement (abdominal X-rays were conducted for both correct duodenal tube placement and to determine the intestinal transit time using Sitzmark capsules as previously described^2^), followed by bowel lavage and FMT from either autologous or allogenic donors according to randomization. Afterward, the subjects started taking either 4 grams of sodium butyrate daily or placebo tablets for 4 weeks, after which the study days were repeated. For 7 days prior to the first and fourth visits, an accelerometer (ActiHeart; CamNTech Ltd., Cambridge, UK) was worn to measure physical activity energy expenditure (PAEE). Fecal samples (for changes in fatty acid metabolism) and 24 h urine samples (for 5-HIAA measurement) were collected preceding the first and fourth visits and brought by the subjects on those visits.

The participants were asked to maintain their habitual physical activity patterns but refrain from heavy exercise before the HIEC procedure days. The participants were allowed to continue their usual diet. All of the participants filled out an online nutritional diary (<https://mijn.voedingscentrum.nl/nl/eetmeter>) to monitor their caloric intake of carbohydrates, fat, protein, and fibers before and after the 4-week intervention.
